# Detection and Characterisation of *Anaplasma marginale* and *A. centrale* in South Africa

**DOI:** 10.3390/vetsci5010026

**Published:** 2018-03-03

**Authors:** Paidashe Hove, Zamantungwa T. H. Khumalo, Mamohale E. Chaisi, Marinda C. Oosthuizen, Kelly A. Brayton, Nicola E. Collins

**Affiliations:** 1 Vectors and Vector-borne Diseases Research Programme, Department of Veterinary Tropical Diseases, University of Pretoria, Pretoria 0110, South Africa; mantungwa98@gmail.com (Z.T.H.K.); marinda.oosthuizen@up.ac.za (M.C.O.); kbrayton@vetmed.wsu.edu (K.A.B.); nicola.collins@up.ac.za (N.E.C.); 2 Biotechnology Platform, Agricultural Research Council, Onderstepoort, Pretoria 0110, South Africa; 3 Research and Scientific Services Department, National Zoological Gardens of South Africa; Pretoria 0001, South Africa; mechaisi@yahoo.co.uk; 4 Department of Veterinary Microbiology and Pathology, Washington State University, Pullman, WA 99164, USA

**Keywords:** bovine anaplasmosis, qPCR, *msp1α* genotyping, Msp1a, Msp1aS

## Abstract

Bovine anaplasmosis is endemic in South Africa and it has a negative economic impact on cattle farming. An improved understanding of *Anaplasma marginale* and *Anaplasma marginale* variety *centrale (A. centrale)* transmission, together with improved tools for pathogen detection and characterisation, are required to inform best management practices. Direct detection methods currently in use for *A. marginale* and *A. centrale* in South Africa are light microscopic examination of tissue and organ smears, conventional, nested, and quantitative real-time polymerase chain reaction (qPCR) assays, and a reverse line blot hybridisation assay. Of these, qPCR is the most sensitive for detection of *A. marginale* and *A. centrale* in South Africa. Serological assays also feature in routine diagnostics, but cross-reactions prevent accurate species identification. Recently, genetic characterisation has confirmed that *A. marginale* and *A. centrale* are separate species. Diversity studies targeting Msp1a repeats for *A. marginale* and Msp1aS repeats for *A. centrale* have revealed high genetic variation and point to correspondingly high levels of variation in *A. marginale* outer membrane proteins (OMPs), which have been shown to be potential vaccine candidates in North American studies. Information on these OMPs is lacking for South African *A. marginale* strains and should be considered in future recombinant vaccine development studies, ultimately informing the development of regional or global vaccines.

## 1. Introduction

A large number of cattle mortalities in South Africa are due to tick-borne diseases, the most important of which are anaplasmosis, babesiosis, and heartwater [1]. Bovine anaplasmosis (or Gall-sickness, as it was formerly known) is a tick-borne disease of ruminants that is caused by microbial pathogens of the genus *Anaplasma* which are obligate, intra-erythrocytic bacteria of the order Rickettsiales and family *Anaplasmataceae* [2,3,4,5,6]. In South Africa, bovine anaplasmosis is endemic in most of the cattle-farming areas [5,7]. In fact, *Anaplasma marginale* is the most prevalent tick-borne pathogen on a global scale, as it is found on all six inhabited continents [6].

Bovine anaplasmosis was first characterised by Sir Arnold Theiler between 1907 and 1910 in South Africa. He observed, in cattle imported from England and infested with ticks in South Africa, the intra-erythrocytic, membrane-bound coccus-like bodies of *A. marginale*, which he described as ‘marginal points’ [2,3,8]. Theiler, through a combination of experimental and epidemiological observations, identified *A. marginale* as the causative agent of bovine anaplasmosis, which had been earlier mistaken as a lifecycle stage of the causative agent of redwater (*Babesia bigemina*). Theiler also identified an organism, which he called *Anaplasma marginale* variety *centrale* (referred to as *A. centrale* hereafter) that generally causes a milder, less virulent form of the disease [5,8]. Infection with *A*. *centrale* confers some cross-protection against *A. marginale*, and it has therefore been employed as a live vaccine from the time it was first developed as such by Theiler in 1910 [5,8].

Anaplasmosis is one of the most economically important diseases of cattle in South Africa [1,5], with symptoms ranging from fever, icterus, inappetence, weight loss, abortion in pregnant cows, and lowered milk production [5,6,9]. It results in significant productivity losses, and, in some cases, mortality [1,9]. Chemical control and treatment measures in South Africa largely involve the use of acaricides to control tick vectors, and long-acting, rickettsicidal tectracyclines, such as the most commonly used oxytetracycline. In South Africa, as in the world over, the effects of tick-borne diseases on animals are often synergistic, where animals are infected with more than one pathogen at a time [1]. Therefore, studies to quantify the losses that are specifically attributable to bovine anaplasmosis are yet to be carried out in South Africa [1,5], and consequently, studies addressing anaplasmosis have been few and far between. In other parts of the world, costs arising from bovine anaplasmosis have been estimated from $US 300–800 million [10]. Furthermore, economic costs attributable to disease burden and control for babesiosis and anaplasmosis together have been approximated at $US 875 million in South America [11] and $US 30.5 million in Australia [12]. Due to the high economic impact, vaccination with *A. centrale* has been deemed to be cost effective for many countries, despite the risk of transmitting emerging pathogens along with the blood-borne vaccine [5].

## 2. Classification of *Anaplasma* Species

Historically, *Anaplasma* spp. have been incorrectly classified as anything from viruses to protozoa [13]. A taxonomic reclassification and reorganization of the genus using genetic analyses [4] provided an invaluable contribution to the systematics of the *Anaplasma* spp. *Anaplasma marginale* is currently regarded as the type species for the genus *Anaplasma* [4], which was expanded to accommodate three species that are reclassified from the genus *Ehrlichia* that invade cells of haematopoietic origin (neutrophils and erythrocytes) in their vertebrate host species. These are *A. phagocytophilum* (formerly known as *Ehrlichia phagocytophila*, *E. equi*, and the agent of human granulocytic ehrlichiosis), *A. bovis* (formerly *E. bovis*) and *A. platys* (formerly *E. platys*). Also included in the genus *Anaplasma* is another species, *A. ovis*, that causes mild to severe disease in sheep, deer and goats. 

Additional species have been reported that are not formally described, including *Anaplasma* sp. (Omatjenne) [formerly *Ehrlichia* sp. (Omatjenne)] [14] and *A. capra* [15]. The name *A. caudatum* was given to an *A. marginale* strain with appendages that also causes bovine anaplasmosis [5]. While this is formally recognised as a separate species, it is thought to be simply a “tailed” strain of *A. marginale*, but has not been studied in great detail [6]. 

*A. centrale* was erroneously classified as a separate species, an error that is attributable Ristic in 1968 [16] who incorrectly stated: “In 1911, Theiler, who first described *A. centrale*, indicated that it was a separate species and thus distinct from *A. marginale*”. While some authors recognised this error and continued to refer to *A. centrale* as a variety or subspecies of *A. marginale*, the organism was listed as a separate species in List No. 15 of new names and new combinations previously effectively published outside the International Journal of Systematic Bacteriology [17] and subsequently in Bergey’s Manual of Systematics of Bacteriology [18]. It is thus referred to as a separate species in many publications. We have recently shown, through sequence analyses of the 16S rRNA gene, *groEL* and *msp4* from several isolates of *A. marginale* and *A. centrale* from around South Africa, that *A. centrale* consistently forms a separate clade from *A. marginale* [19]. These results, when combined with morphological differences, and the differences in Msp1a/Msp1aS gene structure [20], as well as genome architecture [21,22], provide evidence to suggest that *A. centrale* is, in fact, a separate species.

Thus, the current classification of *Anaplasma* species can be considered, as shown below (adapted from [13]), with seven formally recognised species and two others that have not yet been formally described.

**Superkingdom**Bacteria**Phylum**Proteobacteria**Class**Alpha-proteobacteria**Order**Rickettsiales**Family***Anaplasmataceae***Genus***Anaplasma***Species***A. marginale* (type species)
*A. bovis*
*A. caudatum*
*A. centrale*
*A. ovis*
*A. phagocytophilum*
*A. platys*
Not formally described:
*A. capra*
*Anaplasma* sp. (Omatjenne)

## 3. Epidemiology

**Figure 1:**
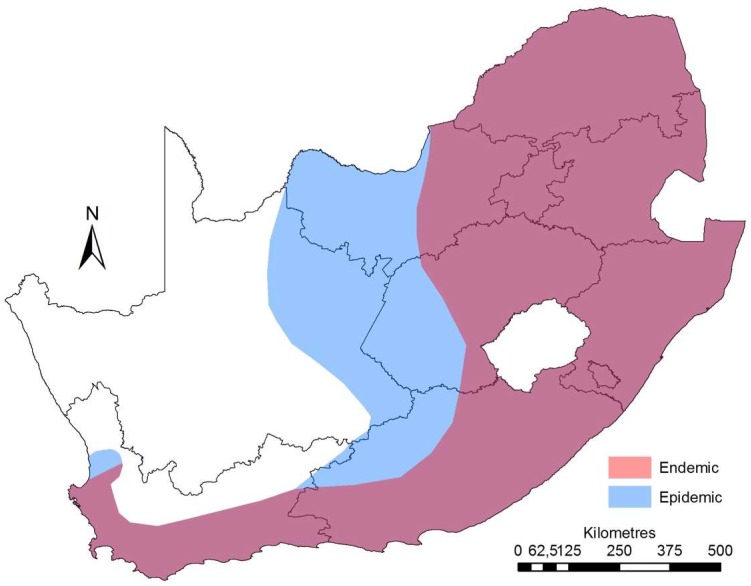
Endemic (red) and epidemic (blue) areas of bovine anaplasmosis disease coverage in South Africa based on historical distribution of vector ticks and areas where the disease has been reported.

Bovine anaplasmosis is endemic in South Africa [1,7,23], although the Northern Cape is considered to be free of the disease [1]. In South Africa, the role played by tick species in anaplasmosis transmission has been poorly studied, and it has long been assumed that the one-host tick, *Rhipicephalus decoloratus* is the main disease vector. This is due to the co-occurrence of this tick and the disease in endemic areas of the country [5] (Figure 1). *Rhipicephalus microplus* is spreading in South Africa and is therefore probably increasing in importance as a vector [24]. Experimental transmission studies have demonstrated transstadial transmission of *A. marginale* by *R. decoloratus*, *R. microplus* and *Rhipicephalus simus*, and experimental intrastadial transmission has been demonstrated for these three tick species, as well as *Rhipicephalus evertsi evertsi* and *Hyalomma marginatum rufipes* [1,5]. *R. simus* has also been shown to transmit *A. centrale* transstadially [25]. More recent data from a study conducted between 2014 and 2017, in which ticks were collected and analysed for *A. centrale* infection, suggests that *A. centrale* is also transmitted by the tick vector, *Rhipicephalus appendiculatus* [26]. However, this is yet to be confirmed by performing transmission studies.

Many antelope and other game species are abundant both in game reserves and farming areas in South Africa, and they are likely to play a role in the epidemiology of anaplasmosis. However, the role of wildlife as reservoir hosts of *Anaplasma* spp. has not been extensively studied. Blesbok (*Damaliscus pygargus phillipsi*), common duiker (*Sylvicapra grimmia*), and black wildebeest (*Connochaetes gnou*) have been experimentally infected with *A. marginale* and *A. centrale*, although the infections were subclinical [5,27]. It has also been shown that blesbok are susceptible to *A. centrale* infection [5]. *Anaplasma* spp. have also been recorded in giraffe (*Giraffa camelopardalis*), sable antelope (*Hippotragus niger*), buffalo (*Syncerus caffer*), and black wildebeest (*Connochaetes gnou*) [5,28]. A more complete understanding of the epidemiology of anaplasmosis is important for both domestic and wild animal health.

The recommended approach to the control of tick-borne diseases in South Africa is the integrated strategic use of acaricides and application of vaccines [1]. Acaricides are expensive, they pose an environmental hazard, and acaricide resistance is rapidly developing among tick populations worldwide [29]. Vaccines available to prevent bovine anaplasmosis, which is caused by *A. marginale*, are currently limited. Infection with *A. centrale* confers cross-protection to *A. marginale*, and *A. centrale* is used in a live blood vaccine in many countries, including South Africa [30]. This vaccine is expensive to produce as live cattle are required, it requires careful maintenance of a cold chain, and carries the risk of unintended introduction of other blood-borne pathogens. The vaccine also does not protect against all field strains of *A. marginale* and can cause severe clinical reactions following vaccination [31].

A recombinant vaccine would circumvent many of the problems that are associated with live blood vaccines. An effective vaccine needs to induce both high IgG2 titres and possess both CD4^+^ T- and B-cell epitopes, which produce robust B- and T-cell memory responses during subsequent *A. marginale* infections [32,33]. Highly promising outer membrane protein (OMP) vaccine candidates have recently been identified primarily from North American strains of *A. marginale* [34,35,36,37,38], but it is not known if these candidates are sufficiently conserved to be broadly useful or if vaccine development based on regional pathogen strains is necessary. The OMPs Am202, Am368, Am854, Am936, Am1041, and Am1096, which have been shown to have between 97 and 100% amino acid identity in strains and isolates from different geographical locations, have recently been assessed as vaccine candidates [39]. This study revealed that, although the four most conserved of these OMPs were consistently recognised by sera from animals vaccinated with outer membrane complexes, OMPs Am854 and Am936 were recognised most consistently. Variation in these OMPs has not yet been examined in South Africa. 

The antibody-sensitive neutralization epitope, Q(E)ASTSS, as first described by Allred et al. [40], and both T-cell (VSSQSDQASTSSQLG) [41] and B-cell (SSAGGQQQESS) [42] epitopes have been described in the N-terminal repeat region of the Msp1a protein. More recently, Omp7–9 have been reported to possess a T-cell epitope (FLLVDDAI/VV) which is conserved between the three A. *marginale* OMPs across strains from America, Australia and Ghana, as well as A. *centrale* [43]. These epitopes have not been examined in South African strains. Therefore, the detection of different *A. marginale* strains in South Africa is necessary in order to assess the variation in vaccine candidate OMPs and to determine if previously identified epitopes are present in South African strains. 

## 4. Detection of *A. marginale* and *A. centrale* in South Africa

A comparison of routinely utilised detection strategies for *A. centrale* and *A. marginale* in South Africa is shown in Table 1. The oldest method is direct detection by light microscopic observation of the parasite in tissue or organ smears after staining with Giemsa and other Romanowksy stains. Giemsa staining of thin blood smears combined with light microscopic examination are routinely used in the detection of *A. marginale* and *A. centrale* in clinical and field samples in South Africa. An earlier, less advanced form of this methodology was employed by Sir Arnold Theiler in the discovery of *A. marginale* and *A. centrale* [2,3,8]. The method is not very sensitive, and is therefore used in conjunction with other assays to confirm infection. In Giemsa-stained thin film blood smears, *A. marginale, A. caudatum* and *A. centrale*, which all infect cattle, appear as dense, deep purple, vacuole-bound, near-circular inclusion bodies, with a diameter ranging from 0.3 to 1 μm. The inclusion bodies are located on the margins of the erythrocytes, except for *A. centrale*, which, as the name implies, has inclusion bodies located centrally [5,6]. Necroscopy accompanied by microscopic examination is also utilized to detect *Anaplasma* in thin films of internal organs such as liver and spleen, along with peripheral blood; smears are stained with dyes, such as toluidine blue, new methylene blue, and acridine orange.

Indirect genus-specific detection of *Anaplasma* species in infected animals is carried out using the following serological tests: major surface protein 5 (Msp5) enzyme-linked immunosorbent assay (ELISA), complement fixation and the card agglutination test [1,5,6,45]. However, the Msp5 ELISA is not able to distinguish between *Anaplasma* spp. Numerous nucleic acid-based assays for the detection of the parasite have been developed and include: conventional polymerase chain reaction (PCR) [48,49], nested PCR (nPCR) [50,53], quantitative real-time PCR (qPCR) [51,52], and a reverse line blot hybridization (RLB) assay [46]. We recently demonstrated the utility of next-generation PCR amplicon sequencing as a tool for detection and analysis of genetic variation in *A. marginale* and *A. centrale* [54]. These tests have been demonstrated to be effective for inter- and intra-species differentiation and for the detection of low levels of rickettsaemia, which cannot be detected in thin blood smears. 

The RLB hybridization assay has been used extensively for the routine screening of cattle and wildlife samples in South Africa and has the ability to detect up to 32 pathogens in one reaction. This technique has been used in the discovery of novel pathogens or genetic variants of known pathogens [55,56]. Its utility lies in its ability to detect *Anaplasma*, *Ehrlichia*, *Babesia* and *Theileria* parasites in a single reaction [46,57], and it is therefore a good screening tool to establish what pathogens might be in a sample. The duplex qPCR test for detection of *A. marginale* and *A. centrale* is a more rapid test than the RLB assay and can be used to confirm the RLB results and for quantification of the infection. We evaluated the performance of three of the nucleic acid-based methods, RLB hybridization, nPCR, and duplex qPCR in the detection of *A. centrale* and *A. marginale* in South African samples [47]. The nPCR assay was shown to give false negative results, due to sequence differences in the internal forward priming region in South African *A. marginale* strains. It was concluded that duplex qPCR is the most sensitive of these three methods, as it detected more *A. marginale* and *A. centrale* positive samples. The duplex qPCR assay has been used in our laboratory for detection and quantification of *A. marginale* and *A. centrale* infections in cattle and wildlife [20,54,55]. Using the qPCR assay, we determined the prevalence of 57% and 17%, respectively, for *A. marginale* and *A. centrale* infections in South African cattle, as well as a co-infection rate of 15%. These studies [20,54] suggest that *A. centrale* is circulating naturally in South African cattle, as it was found in non-vaccinated cattle and wild animals. 

## 5. Genetic Diversity of *A. marginale* and *A. centrale* in South Africa

### 5.1. msp1α Genotyping of A. marginale

**Figure 2:**
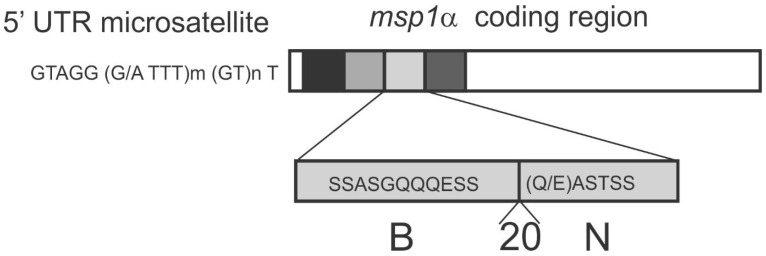
A schematic diagram of the *msp1α* gene. The tandem repeats are shown as grey boxes, with each shade representing a different repeat. The microsatellites in the 5' untranslated region (UTR) used in a second genotyping system are shown. The B-cell (B) and neutralization sensitive (N) epitopes, and amino acid 20, found to be important for binding to tick cell extracts, are shown on an enlarged protein repeat.

Genotyping efforts using the *msp1α* gene are well advanced in DNA-based strain differentiation of *A. marginale* strains [7,58,59]. *msp1α* is a single copy gene encoding major surface protein 1a (Msp1a). The gene can be used to characterise strain differences due to variations in the number and sequence of tandem repeats at the 5′ end of the gene [40,60] (Figure 2). A complex system has been developed in which the Msp1a repeats are named alphanumerically, in order to distinguish sequence variants, leading to *msp1α* genotypes being described as, for example, J/B/B (the St. Maries strain) or A/B/B/B/B/B/B/B (the Florida strain) [58]. The current, most widely used PCR-based *msp1α* genotyping protocol is based on the PCR methodology, as described by Lew et al. [53] and de la Fuente et al. [61]. *msp1α* genotyping has elucidated the genotypic variation found in *A. marginale* strains in virtually all the regions of the world that are plagued by anaplasmosis, including South Africa [7,23,54], Asia [15,62], Australia [53], Europe [59,63], South America [64,65] and North America [66,67]. A tool was recently developed to provide analytics for Msp1a repeats which also provides databasing capabilities [68]. 

*A. marginale* strains present in different herds show variation in Msp1a repeat structure and it is thought that this can be indicative of sequence variation in other antigenically significant proteins [58,69]. Msp1a has also been shown to contain B-cell and neutralization sensitive epitopes, and, in the repeats, amino acid 20 is thought to be important for binding to tick cells [40,42] (Figure 2).

A parallel genotyping system, based on applying a formula to the number of the microsatellite repeats found between the Shine-Dalgarno sequence (GTAGG) and the initiation codon (ATG) sequence upstream of the *msp1α* coding sequence has been described [63]. However, this genotyping scheme is used much less frequently, and the significance of the genotypes remains unclear.

The first study to examine Msp1a in the South African context demonstrated *msp1α*-based genetic diversity in *A. marginale* strains from the Free State province, and identified Msp1a repeats that are similar to repeats identified in strains from the United States strains, as well as repeats unique to South Africa [7]. Furthermore, 42% of the Msp1a repeats were shared between South African strains and those from South America, North America, and Europe.

Another study used *msp1α* sequence data to examine the epidemiology and genetic diversity of *A. marginale* strains in South Africa and suggested mechanisms for the evolution of *A. marginale* [23]. This study found a 65–100% prevalence of *A. marginale* in different provinces, along with the associated Msp1a genetic diversity in each province. This diversity was highlighted by the 23 novel Msp1a tandem repeats found in South African *A. marginale* strains, which are likely to have evolved from tandem repeat 4. Interestingly, it was also shown that genetic diversity in the highly variable Msp1a was evolving under both positive and negative selection pressure in the South African *A. marginale* population. Using a bioinformatics approach, the authors also showed that Msp1a contains B- and T-cell epitopes, with serine residues that are highly conserved in the repeat region and are thought to be important for the adhesion function of the Msp1a protein. This suggests that Msp1a is a possible vaccine candidate, despite its highly variable amino acid residues. The same B- and T-cell epitopes were also identified in a more recent South African study [54]. 

We recently assessed South African Msp1a genetic diversity and found 36 novel Msp1a repeats that were contributing to a total of 99 described in the country to date [54]. These 99 repeats are configured to make up 190 genotypes, suggesting that strain variation across South Africa is prevalent. However, caution needs to be taken in interpreting this genetic variation as assessment of genetic diversity using *msp1α* genotypes is based on a single genetic locus, and the inference that this locus is a surrogate reporter for more widespread genomic variation is based on a single study [58].

### 5.2. msp1aS Genotyping of A. centrale

We developed a novel genotyping system for *A. centrale* based on the Msp1aS protein, a homolog of *A. marginale* Msp1a [20]. The genotyping methodology is similar to *msp1α* genotyping in *A. marginale*, the only difference being that the repeats in Msp1aS are larger than the repeats in Msp1a. A total of 47 Msp1aS repeats were identified in South African cattle, wildebeest, and buffalo, representing 32 *A. centrale* genotypes, which were described for the first time and are distinguishable from the vaccine strain. The study revealed genetic diversity of *A. centrale* strains in cattle and wildlife, and suggested that wildlife could be reservoirs of *A. centrale* infection [20]. The study also showed that Msp1aS could be utilised as a genetic marker for diversity analysis in *A. centrale.*

Both of our recent studies examining *A. marginale* and *A. centrale* in South Africa [20,54] have used the program RepeatAnalyzer [68] to identify, curate, map, and analyse Msp1aS (*A. centrale*) and Msp1a repeats (*A. marginale*). These studies reveal the urgent need for a centralized online repeat genotype/strain repository along with the development of a unified nomenclature for *A. marginale* and *A. centrale*.

## 6. Conclusions

The South African studies that are outlined in this mini-review, along with other studies elsewhere in the world, highlight the variety of assays employed in detection and evaluation of genetic diversity in *A. marginale* and *A. centrale*. While nucleic acid based assays have been widely used in South Africa, these have to be used judiciously and in conjunction with direct methodologies, such as tissue and organ staining, combined with light microscopy. The *A. marginale msp1α* genotyping studies carried out in South Africa confirm that *A. marginale* is endemic in the country and is a genetically diverse organism that is continuously evolving. Genetic diversity of *A. marginale* and the corresponding variation in OMP genes of immunogenic importance, need to be considered when developing a recombinant vaccine, which is likely to be the future of *A. marginale* control. 

## Figures and Tables

**Table 1:** Comparison of diagnostic assays currently in use in South Africa for detection of *A. marginale* and *A. centrale.*

Assay	Cost per sample (South African Rand - R)	Average throughput time	Comments on assay sensitivity	Technical skills & expensive equipment needed?
Light microscopic examination of Giemsa-stained smears [5,44]	R113	3 days	Low (10^6^ *A. marginale*- infected erythrocytes per ml of blood) Best used during acute phase of infection	Low to Medium No
Msp5 competitive ELISA (cELISA) [5,45]	R140	4 days	Low to Medium Results in false negatives Detects *Anaplasma* to genus level only	Medium to High Yes
Reverse line blot (RLB) hybridisation [46,47]	R445	3 days	Medium to high Similar to PCR & higher than nPCR, but lower than qPCR	Medium to High Yes
Conventional PCR [48,49]	R250	2 days	Medium Similar to RLB	Medium to High Yes
Nested PCR [47,50]	R350	3 days	Medium Fails to detect genetic variant sequences leading to false negatives Less sensitive than RLB & qPCR	Medium to High Yes
Duplex quantitative real-time PCR (qPCR) [47,51,52]	R430	2 days	High (30 *Anaplasma-* infected erythrocytes per ml of blood) Detects parasites at very low levels Most sensitive test available in South Africa	Medium to High Yes
